# Comparison of clinical and radiological characteristics of inflammatory and non-inflammatory Rathke cleft cysts

**DOI:** 10.1007/s11604-024-01641-0

**Published:** 2024-08-20

**Authors:** Shu Matsushita, Taro Shimono, Hiroyuki Maeda, Taro Tsukamoto, Daisuke Horiuchi, Tatsushi Oura, Kenichi Ishibashi, Hirotaka Takita, Hiroyuki Tatekawa, Natsuko Atsukawa, Takeo Goto, Yukio Miki

**Affiliations:** 1 Department of Diagnostic and Interventional Radiology, Graduate School of Medicine, Osaka Metropolitan University, 1-4-3, Asahi-machi, Abeno-ku, Osaka, 545-8585 Japan; 2 Department of Neurosurgery, Osaka City General Hospital, 2-13-22, Miyakojima-Honndori, Miyakojima-ku, Osaka, 534-0021 Japan; 3 Department of Neurosurgery, Graduate School of Medicine, Osaka Metropolitan University, 1-4-3, Asahi-machi, Abeno-ku, Osaka, 545-8585 Japan

**Keywords:** Pituitary, Rathke’s cleft cyst, Inflammation, MRI

## Abstract

**Purpose:**

Rathke cleft cysts are commonly encountered sellar lesions, and their inflammation induces symptoms and recurrence. Cyst wall enhancement is related to inflammation; however, its range and frequency have not yet been investigated. This study aimed to investigate the clinical and radiological differences between inflammatory and non-inflammatory Rathke cleft cysts.

**Methods:**

Forty-one patients who underwent cyst decompression surgery for Rathke’s cleft cysts between January 2008 and July 2022 were retrospectively analyzed. Based on the pathological reports, patients were divided into inflammatory and non-inflammatory groups. Clinical assessments, endocrinological evaluations, cyst content analysis, and imaging metrics (mean computed tomographic value, maximum diameter, mean apparent diffusion coefficient [ADC] value, and qualitative features) were analyzed. Receiver operating characteristic curve analysis was performed, to determine ADC cutoff values, for differentiating inflammatory group from non-inflammatory group.

**Results:**

Totally, 21 and 20 cases were categorized into the inflammatory and non-inflammatory groups, respectively. The inflammatory group displayed a higher incidence of central diabetes insipidus (arginine vasopressin deficiency) (*p* = 0.04), turbid cyst content (*p* = 0.03), significantly lower mean ADC values (*p* = 0.04), and more extensive circumferential wall enhancement on magnetic resonance imaging (MRI) (*p* < 0.001). In the inflammatory group, all cases revealed circumferential wall enhancement, with some exhibiting thick wall enhancement. There were no significant differences in other radiological features. The ADC cutoff value for differentiating the two groups was 1.57 × 10^−3^ mm^2^/s, showing a sensitivity of 81.3% and specificity of 66.7%

**Conclusion:**

Inflammatory Rathke cleft cysts tended to show a higher incidence of central diabetes insipidus and turbid cyst content. Radiologically, they exhibited lower mean ADC values and greater circumferential wall enhancement on MRI.

## Introduction

Rathke cleft cysts (RCCs) are the most commonly encountered sellar lesions by radiologists. In a cadaveric study small RCCs were present in 21% specimens among all incidental pituitary lesions [1]. When RCCs grow, they can cause a mass effect on surrounding structures such as the pituitary gland, hypothalamus, and optic chiasm, leading to symptoms such as headache, visual loss, and endocrine dysfunction [2].

Inflammation in RCC is clinically problematic because it can easily cause symptoms and recurrence [2]. Squamous metaplasia and stratification of the epithelium are the pathological changes consequent to chronic inflammatory reaction [3]. Long-term recurrence rate of RCCs is associated with the squamous metaplasia, and monitoring is recommended for at least 5 years [4]. Owing to inflammation, the cyst capsule may adhere to surrounding structures; consequently, surgeons should be cautious during surgical procedures compared with handling RCC without inflammation [5]. Notably, cysts have shrunk with glucocorticoid administration in several cases [6, 7].

The relationship between inflammation and radiological findings in RCC remains unclear. Typical radiological findings of RCCs are oval-shaped cystic lesions in the sella between anterior and posterior pituitary lobes [8]. Usually, the cyst wall is not enhanced after contrast-media administration [9, 10]. However, in daily practice, some cases are difficult to diagnose as RCCs because of wall calcification or enhancement [11, 12]. Kim et al. [13], demonstrated that 42.5% of 40 symptomatic RCCs exhibited inflammatory cell infiltration; however, their radiological findings were not detailed. Zhong et al. [14] noted pathological inflammation in 7/12 cases that showed marked wall enhancement. However, the range and frequency of wall enhancement associated with RCC inflammation were not described. Despite the differences in symptoms and recurrence frequency, radiological differences between RCCs with and without inflammation have not been investigated.

Evaluating the presence of inflammation in RCCs using imaging helps to determine treatment strategies, such as when to perform surgery and how to manage perioperative care. Therefore, this study aimed to determine the clinical and radiological differences between RCCs with and without inflammation.

## Methods

### Participants

This retrospective study was approved by the Institutional Review Board of our institution, and the requirement for written informed consent was waived owing to the retrospective nature of this study. Patients’ anonymity was maintained. Inclusion criteria were as follows: patients who underwent cyst decompression surgery at our institution or an affiliated hospital between January 2008 and July 2022, those radiologically or pathologically diagnosed with RCC, and those with available preoperative images. Individuals who had undergone surgery for sellar lesions before surgery for RCC were excluded. Finally, 41 patients were enrolled in this study.

### Definition of inflammatory RCC

We reviewed the pathological reports of all included patients; of which, 21 cases with inflammatory cell infiltration, squamous epithelial metaplasia, and granulation tissue formation were defined as inflammatory RCCs [3, 15].

### Clinical and laboratory evaluation

Chief complaint, and physiological and endocrinological symptoms at the time of admission for surgery were obtained from the medical records. Preoperative endocrinological evaluation was performed in all patients and consisted of basal serum hormone. Subsequently, dynamic endocrine tests were performed in 26 patients. Water deprivation tests were performed on patients suspected of having diabetes insipidus (arginine vasopressin deficiency) [16, 17]. Results of dynamic endocrine and water deprivation tests were evaluated by endocrinologists. Cyst contents were also checked based on surgical findings and classified as clear, turbid, or others.

### Computed tomography and magnetic resonance imaging examination techniques

All patients underwent computed tomography (CT) without contrast-enhance material. CT images with a section thickness of 1–5 mm were obtained in the axial, coronal, and sagittal multiplanar reformatted planes without an intersection gap using various models of multidetector row CT scanners (Aquilion One: Canon Medical Systems, Otawara, Japan; Aquilion, Xvigor, Asteion: Toshiba Medical Systems, Tokyo, Japan; LightSpeed VCT: GE Healthcare, Milwaukee, WI, USA; SOMATOM Sensation64: Siemens, Erlangen, Germany). Various imaging methods have been used owing to updates and changes in 1.5 T (Achieva: Philips Healthcare, Best, the Netherlands; Avanto: Siemens; Signa: GE Healthcare) and 3 T (Achieva: Philips Healthcare; Skyra: Siemens) magnetic resonance (MR) scanners during the long study period. Unenhanced T1WI (repetition time (TR)/echo time (TE) range, 400–550/12–16 ms; field of view (FOV), 18–19 cm; slice thickness, 1–5 mm) was performed in the sagittal (39 patients), coronal (32 patients), and axial (16 patients) planes.T2WI (TR/TE range 2300–4000/85–100 ms; FOV, 18–23 cm; slice thickness, 3–5 mm) was performed in the sagittal (37 patients), coronal (22 patients), and axial (32 patients) planes. Contrast-enhanced T1WI (TR/TE range 3.9–590/1.7–16 ms; FOV, 18–25 cm; slice thickness, 1–3 mm) after an intravenous injection of contrast medium (0.1 mmol/kg of body weight) was also acquired, except for one who was pregnant, in the sagittal (40 patients), coronal (40 patients), and axial (37 patients) planes. Three-dimensional constructive interference in steady state images (TR/TE range 1400–1500/230–250 ms; FOV, 16–19 cm; slice thickness, 1.0–1.5 mm) were obtained from 24 patients. Diffusion-weighted images (TR/TE range 3000–8800/61–90 ms; FOV, 22–23 cm; slice thickness, 2.5–5 mm) were obtained for 37 patients. Apparent diffusion coefficient (ADC) maps were available for 32 patients. The *b* values were 0 and 1000 s/mm^2^.

### Quantitative image analysis

Mean CT value (H.U.), maximum diameter (mm), and mean ADC value (10^–6^ mm^2^/s) were measured by a single radiologist with 4 years of experience. Other three radiologists with 6, 6, and 12 years of experience validated these measurement methods. CT and ADC values were measured by drawing a small oval-shaped (5–10 mm^2^) region of interest (ROI) in the cyst. ROIs were placed inside the superior part of the cyst in the axial plane to minimize paranasal artifacts. When the boundary of the cyst was unclear, or the cyst was strongly distorted due to artifacts, ROI placement was decided by consensus among the other three radiologists. Maximum diameter was the largest anteroposterior, superoinferior, and transverse dimensions.

### Qualitative image analysis

All images were retrospectively reviewed by three radiologists with 6, 6, and 12 years of experience, who were aware of the diagnosis of RCC, but were blinded to the presence of inflammation. The following imaging features were evaluated individually by three radiologists using CT/MRI: calcification, location, superior extension, loculation, shape, lateral extension, midline/off-midline, homogeneity, signal intensity (SI) on T1 and T2WI, parasellar T2 dark sign, fluid–fluid level, intracystic nodule, posterior pituitary bright spot, damming-up phenomenon (ectopic posterior pituitary bright spot at the pituitary stalk), mucosal thickening, circumferential wall enhancement, enhancement in wall thickness, stalk thickening, basisphenoid enhancement, and dural thickening. In case of discrepancy, a final decision was made by reaching a consensus.

Calcification was classified as absent, present on the wall, or present within the cyst. Cyst location was categorized as intrasellar, intra and suprasellar, or suprasellar. Superior extensions were those below the optic chiasm, compressing the optic chiasm, or compressing the third ventricle. Compression of the third ventricle was considered if the third ventricle floor was indented [18]. Loculation was classified as unilocular or multilocular. Shapes were ovoid snowman-like, superiorly or inferiorly lobulated. Snowman shape was a figure of eight-like shape and a superiorly or inferiorly lobulated shape was two or more lobes in the suprasellar or intrasellar compartment, respectively. Lateral extension was an extension within or beyond the lateral margin of the cavernous part of the internal carotid artery. Off-midline location was lateralization of the lesion in the sella turcica or stalk deviation by the lesion [19]. SI on T1 and T2WI was hyper/iso/hypointense compared to that of the gray matter, focusing on the area occupying the largest portion of the cyst. Parasellar T2 dark sign was defined as low SI of basisphenoid bone marrow along the sella wall on coronal and sagittal planes of T2WI [20]. Fluid–fluid level was defined as different intensities separated by a linear border on either T1WI or T2WI. An intracystic nodule was a nodule exhibiting various SI on T1 and T2WI without enhancement after contrast-medium administration. Posterior pituitary bright spot was high SI of the normal posterior pituitary lobe on unenhanced sagittal T1WI [21]. Damming-up phenomenon was high SI of the stalk on unenhanced T1WI [22]. Mucosal thickening was recorded when paranasal mucosal thickening was identified on any image. Cyst wall enhancement was classified as none, less than a half, or more than a half of the entire circumference. Enhancement in wall thickness was classified as thin (< 2 mm), or thick (≥ 2 mm) [18, 23]. Anterior pituitary lobe was identified as a nodular or flattened structure, with a slight thickness projecting externally from the cyst wall. Care was taken not to include the anterior lobe as a part of enhancing wall. Stalk thickening was defined as measuring > 3.5 mm or > 2.5 mm at the proximal or distal portion in its transverse diameter, respectively [24, 25]. Basisphenoid enhancement was the basisphenoid bone marrow enhancement on coronal and sagittal plane of fat-saturated contrast enhanced T1WI [26]. Dura matter thickening along the clivus or anterior skull base was defined as dural thickening.

### Statistical analysis

All statistical analyses were performed using the R software (version 4.0.2, 2020; R Foundation for Statistical Computing; http://www.r-project.org/). The Mann–Whitney *U*-test was used to evaluate differences in the distribution of age, mean CT and ADC values, and maximum diameter between inflammatory and non-inflammatory RCCs. Fisher’s exact test was used to compare the distribution of sex, frequency of symptoms, endocrine abnormalities, cyst contents, and other imaging features between inflammatory and non-inflammatory RCCs.

The inter-rater agreements for imaging findings other than CT and ADC values, and maximum diameter were evaluated using Fleiss *κ* coefficient. The Fleiss’ κ coefficient was defined as poor agreement, ≤ 0.2; fair agreement, 0.2–0.4; moderate agreement, 0.4–0.6; good agreement, 0.6–0.8; and very good agreement, > 0.8 [27]. Receiver operating characteristic (ROC) curve analysis was performed to determine ADC cutoff values for differentiating inflammatory RCCs from non-inflammatory RCCs. Statistical significance was set at *p* < 0.05.

## Results

### Clinical and laboratory findings

Clinical and laboratory findings are summarized in Table 1. Age distribution was not significantly different between inflammatory and non-inflammatory RCCs groups. Although there was a predominance of female patients, the sex distribution was not significantly different between the inflammatory and non-inflammatory RCCs. The frequency of each symptom, such as headache, visual deficit, menstrual abnormality, short stature, diabetes insipidus, and lactation, was not significantly different between the groups. Among the endocrine deficiencies, central diabetes insipidus was a significantly frequent finding in inflammatory RCCs (*p* = 0.04). The distribution of the cyst content exhibited a statistically significant difference (*p* = 0.03).

**Table 1:** Summary of clinical and laboratory features between inflammatory and non-inflammatory RCCs

	Inflammatory RCC (n = 21)	Non-inflammatory RCC (n = 20)	*p* value
Mean age (range, SD)	44 (6–74, ± 21.0)	33 (7–70, ± 19.1)	0.09
Gender, male: female	4:17	6:14	0.48
Symptoms n (%)			
Headache	8 (38)	9 (45)	0.76
Visual deficit	15 (71)	12 (60)	0.52
Menstrual abnormality	3 (14)	4 (20)	0.70
Short statue	0 (0)	1 (5)	0.49
Diabetes insipidus	7 (33)	2 (10)	0.13
Lactation	0 (0)	1 (5)	0.49
Endocrine deficiencies (%)			
Adrenocorticotrophic axis	9 (43)	3 (15)	0.09
Gonadotroph axis	9 (43)	3 (15)	0.09
Thyrotrophic axis	6 (29)	2 (10)	0.24
Growth hormone axis	8 (38)	7 (35)	1.0
Central diabetes insipidus	7 (33)	1 (5)	0.04*
Hyperprolactinemia	12 (57)	7 (35)	0.21
Cyst contents			0.02*
Clear	2	9	
Turbid	12	6	
Others	7	5	

*RCC* Rathke cleft cysts, *SD* standard deviation

^*^Statistically significant

### Imaging findings

All ROI placements for measuring CT and ADC values were confirmed by three radiologists. ADC maps were available for all 32 patients. However, because of paranasal artifacts in four of these cases, placing an ROI in the cyst was difficult; thus, mean ADC values were evaluated in 28 cases (inflammatory RCC, 12 cases; non-inflammatory RCC, 16 cases).

The sphenoid sinus was postsellar in 20 cases; thus, the parasellar T2 dark sign and basisphenoid enhancement were evaluated in the remaining 21 cases (inflammatory: non-inflammatory RCC, 7:14 cases) [28].

**Fig. 1:**
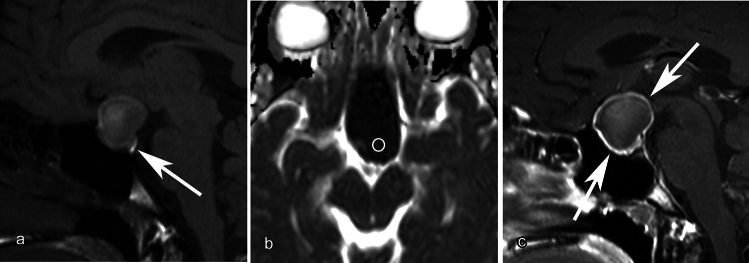
A 20-year-old female with an inflammatory Rathke cleft cyst who presented with visual deficit. **a** An ovoid unilocular lesion is seen compressing the optic chiasm and posterior pituitary bright spot is preserved on sagittal T1WI (white arrow). The cyst displays heterogeneous signal intensity appearing slightly hyperintense on T1WI. **b** The mean ADC value is 0.45 × 10^−3^ mm^2^/s. **c** Most of the cyst wall is circumferentially enhanced on the postcontrast fat-saturated sagittal T1WI (white arrows)

**Fig. 2:**
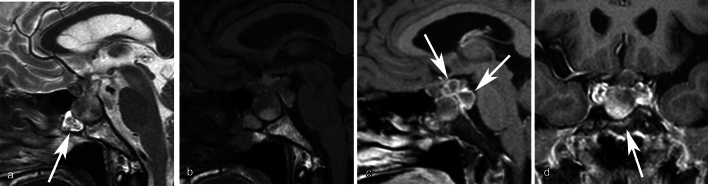
A 59-year-old female with an inflammatory Rathke cleft cyst who presented with visual deficit. **a** A multilocular cystic lesion with superior lobulation is compressing the optic chiasm on sagittal T2WI. The arrow indicates mucosal thickening of the sphenoid sinus. **b** The cyst fluid shows hyperintensity on sagittal T1WI. **c** Postcontrast fat-saturated sagittal T1WI demonstrates ring enhancement of each locules (white arrows). **d** Postcontrast fat-saturated coronal T1WI shows diffuse intrasellar basisphenoid bone marrow enhancement (white arrow). The mean ADC value is 0.41 × 10^−3^ mm^2^/s (not shown)

**Fig. 3:**
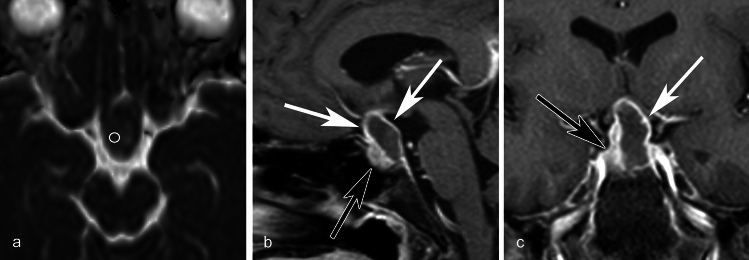
A 54-year-old female with an inflammatory Rathke cleft cyst who presented with visual deficit and diabetes insipidus. **a** The mean ADC value is 1.08 × 10^−3^ mm^2^/s. **b** Fat-saturated postcontrast sagittal T1WI shows an ovoid suprasellar lesion compressing the optic chiasm with more than half circumferential enhancement of the wall (white arrows). The anterior pituitary lobe is displaced anteriorly (black arrow).**c** Fat-saturated postcontrast coronal T1WI shows partially thick enhancement of the wall (white arrow). The anterior pituitary lobe is displaced to right side of the cyst (black arrow)

**Fig. 4:**
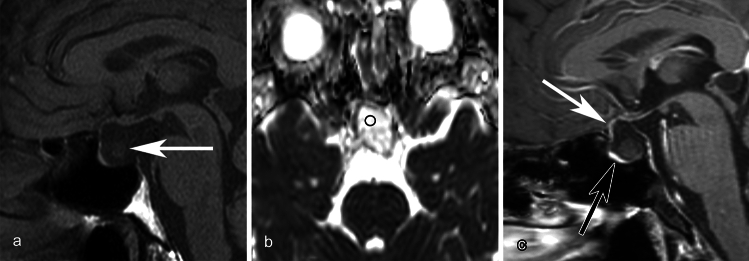
A 47-year-old male with a non-inflammatory Rathke cleft cyst who presented with headache. **a** An ovoid unilocular lesion is seen compressing the optic chiasm on sagittal T1WI. The intracystic nodule shows slightly high-signal intensity compared to the cyst fluid (white arrow). **b** The mean ADC value is 2.30 × 10^−3^ mm^2^/s. **c** Fat-saturated postcontrast sagittal T1WI shows marked enhancement of the anterior pituitary lobe displaced to anteroinferior side of the cyst (black arrow). Anterosuperior part (less than half) of the cyst wall is enhanced (white arrow)

**Fig. 5:**
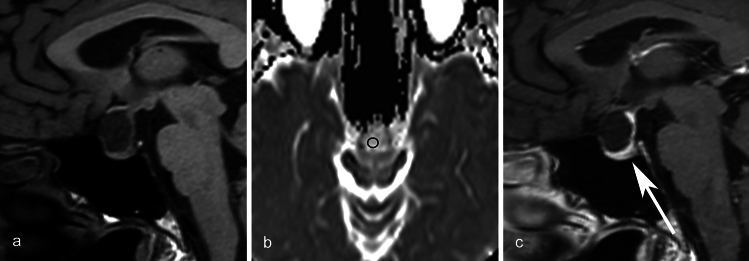
A 47-year-old female with a non-inflammatory Rathke cleft cyst who presented with headache, visual deficit, and menstrual abnormality. **a** An ovoid unilocular lesion is seen compressing the optic chiasm on sagittal T1WI. **b** The mean ADC value is 1.84 × 10^−3^ mm^2^/s. **c** Fat-saturated postcontrast sagittal T1WI shows anterior pituitary lobe displaced to inferior side of the cyst (white arrow). Linear enhancement present posterior to the cyst is considered as the pituitary stalk

Mean CT values and maximum diameter indicated no significant differences between inflammatory and non-inflammatory RCCs, whereas mean ADC values of the inflammatory RCCs were significantly lower than those of non-inflammatory RCCs (*p* = 0.04). Circumferential wall enhancement was significantly broader in inflammatory RCCs (*p* < 0.001). In the inflammatory group, some degree of wall enhancement was observed in all cases, and some exhibited thickened wall enhancement. All cases without wall enhancement comprised the non-inflammatory group. In the non-inflammatory group, some cases exhibited wall enhancement, but all had thin walls. Although basisphenoid enhancement was frequently observed in inflammatory RCCs, the difference between inflammatory and non-inflammatory RCCs was not significant (*p* = 0.09). No other imaging findings exhibited significant differences (*ps* > 0.13). Detailed findings and inter-rater agreements are presented in Table 2. Representative images of inflammatory and non-inflammatory RCCs are depicted in Figs 1, 2, 3, 4, 5.

**Table 2:** Imaging findings between inflammatory and non-inflammatory RCCs

	Inflammatory RCC (n = 21)	Non-inflammatory RCC (n = 20)	*p* value	Fleiss *κ* coefficient
Mean CT value (range, SD; Hounsfield unit)	25.2 (1.2–47, ± 12.2)	32.1 (5–60, ± 16.6)	0.13	
Maximum diameter (range, SD; mm)	21 (14–32, ± 5.5)	20 (8–40, ± 7.5)	0.35	
Mean ADC value (range, SD; 10^–3^ mm^2^/s)^a^	1.29 (0.35–2.57, ± 0.77)	1.89 (0.42–2.73, ± 0.66)	0.04*	
Calcification			0.37	0.57
Absent	20	19		
Wall	0	1		
Inside the cyst	1	0		
Location			0.46	0.41
Intra	0	1		
Intra + supra	20	17		
Supra	1	2		
Superior extension			0.78	0.63
Below optic chiasm	4	3		
Compressing optic chiasm	16	15		
Compressing third ventricle	1	2		
Unilocular/multilocular	17/4	19/1	0.34	0.54
Shape			0.38	0.62
Ovoid	8	9		
Snowman-like	9	10		
Superior lobulation	3	0		
Inferior lobulation	1	1		
Lateral extension, yes/no	0/21	0/20	1.0	1.0
Midline/off-midline	18/3	15/5	0.45	0.43
Homogeneous/inhomogeneous	7/14	7/13	1.0	0.76
Signal intensity on T1WI			0.25	0.80
Low	8	9		
Iso	2	5		
High	11	6		
Signal intensity on T2WI			0.63	0.64
Low	4	3		
Iso	4	2		
High	13	15		
Parasellar T2 dark sign, yes/no^b^	2/5	1/13	0.25	0.64
Fluid–fluid level, yes/no	0/21	0/20	1.0	1.0
Intracystic nodule, yes/no	11/10	9/11	0.76	0.90
Posterior pituitary bright spot, yes/no	6/15	9/11	0.34	0.41
Damming-up phenomenon, yes/no	0/21	1/19	0.49	1.0
Mucosal thickening, yes/no	1/20	0/20	1.0	1.0
Circumferential wall enhancement			< 0.001*	0.66
Absent	0	10		
Less than a half	4	7		
More than a half	17	2		
Enhanced wall thickness, thick (≥ 2 mm)/thin (< 2 mm)	6/15	0/9	0.14	0.73
Stalk thickening, yes/no	6/15	4/15	0.72	0.68
Basisphenoid enhancement, yes/no^b^	3/4	1/13	0.09	0.38
Dural thickening, yes/no	1/20	0/19	1.0	1.0

*RCC* Rathke cleft cysts, *SD* standard deviation

^a^evaluated in 12 and 16 cases of inflammatory and non-inflammatory RCCs, respectively

^b^evaluated in seven and 14 cases of inflammatory and non-inflammatory RCCs, respectively

^*^ Statistically significant

**Fig. 6:**
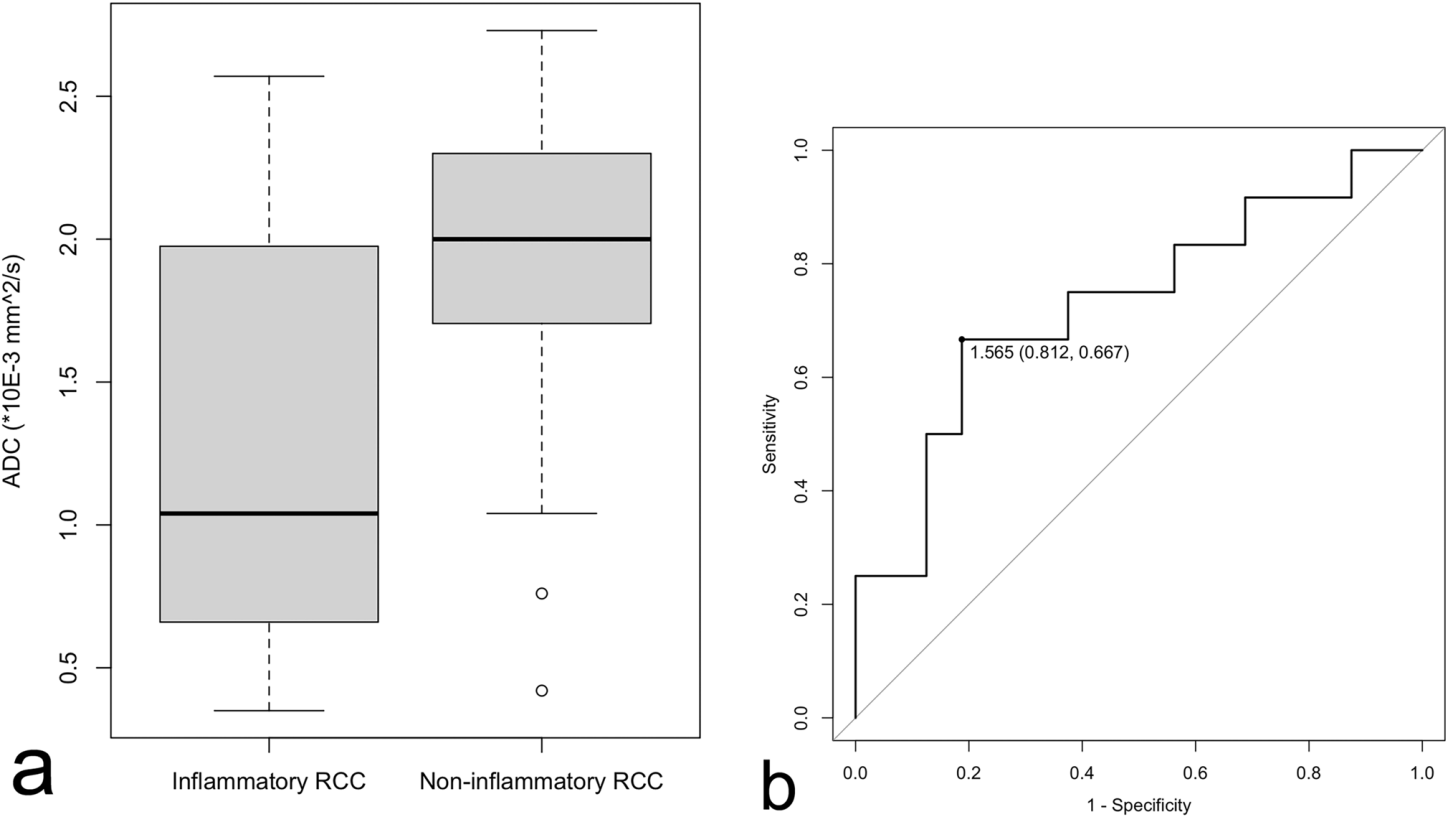
**a** Box and whisker plot of mean apparent diffusion coefficient (ADC) value among inflammatory and non-inflammatory Rathke cleft cysts. **b** Receiver operating characteristic (ROC) curves for mean ADC value. The area under the ROC curve is 0.73

The box-plot graphic evaluation of mean ADC value distribution among inflammatory and non-inflammatory RCCs are shown in Fig. 6a. The cutoff value determined by the ROC curve for differentiation of the inflammatory RCCs from non-inflammatory RCCs was 1.57 × 10^−3^ mm^2^/s, sensitivity was 81.2% and specificity 66.7% (Fig. 6b).

## Discussion

We investigated 41 cases of RCCs and compared clinical and radiological findings of inflammatory (21) and noninflammatory RCCs (20). Endocrine examination revealed central diabetes insipidus as a significant finding in patients with inflammatory RCCs. Surgical findings indicated turbid cyst content in inflammatory RCCs. In the inflammatory group, some degree of wall enhancement was observed in all cases. All cases with thick wall enhancement consisted of inflammatory group. Mean ADC value and circumferential wall enhancement range differed significantly between inflammatory and non-inflammatory RCCs in the radiological findings.

Alsavaf et al. [29] observed preoperative pituitary hypofunction in patients with inflammatory RCC. In our study, we observed a frequent reduction in each pituitary function associated with inflammatory RCC, aligning with previous research findings. However, among these pituitary functions, only central diabetes insipidus demonstrated statistically significant differences. This could be related to the lower incidence of posterior pituitary bright spots on MRI scans among the inflammatory RCC group, even though it was not significant. The sample size and location of the cysts may have affected our results. Diabetes insipidus associated with RCC may be caused by impairment of the stalk and posterior lobe owing to mechanical or inflammatory responses [21]. Up to 37% patients with RCC reportedly experience preoperative diabetes insipidus [30–34]. Patients with diabetes insipidus show relatively low rate of improvement compared with those with hyperprolactinemia [31, 34]. Clinicians should be aware of diabetes insipidus in patients with suspected RCC and discuss appropriate timing of intervention.

The cyst content in RCC typically appears clear and resembles cerebrospinal fluid, characterized by a low protein content [2]. However, the cyst content may display a thick, mucoid substance composed of cholesterol and proteins, commonly seen among patients who exhibit symptoms [2]. Among our cohort, the cyst fluid tended to be turbid in the inflammatory RCC group. This could be attributed to the exudate caused by inflammation.

CT images were not useful in distinguishing whether inflammation is present or absent in RCC. Most RCC have a low density on CT, whereas some have mixed high and low attenuation [35]. The precise CT value of the cyst content has been reported up to 105 Hounsfield units in case reports [36, 37]. The limited availability of reports concerning CT values may be attributed to the unreliability of measurements, which are often caused by artifacts from air and bone. In our data, mean CT value was lower in the inflammatory group, though the cyst fluid tended to be turbid in this group. However, the meaning of this result is not clear. Although calcification in RCC is related to inflammation and degeneration [11], the frequency of calcification was not significant in our inflammatory RCC group. This might be because calcification in RCC is quite rare.

Only a few studies have used DWI in pituitary disease analysis [38, 39]. Kunii et al. [39] reported the ADC value of RCC as 2.12 ± 0.29 × 10^–3^ mm^2^/s, which is higher than that of craniopharyngioma and hemorrhagic pituitary neuroendocrine tumor (PitNET), formerly known as pituitary adenoma [40]. They utilized single-shot fast spin echo method to avoid image distortion of skull base and sinonasal cavities. Our DWI data were obtained using the echo-planar method in axial plane, which may have affected the study results. However, we eliminated the effects of artifacts by excluding cases with no visible lesions on DWI and by placing the ROI on the superior part of the cyst. Low ADC value of the inflammatory RCC group may reflect diffusion restriction of the exudative cyst content. Radiologists should be aware that ADC value can be relatively low in symptomatic RCC. Studies comparing the ADC value of inflammatory RCC with that of other cystic sellar pathologies, including craniopharyngioma, cystic PitNET, and arachnoid cyst are needed.

Our cohort demonstrated a more extensive wall enhancement in the inflammatory group, which is consistent with the findings of previous studies. Additionally, all cases exhibiting thick wall enhancement were classified under the inflammatory group, while those lacking wall enhancement were categorized as part of the non-inflammatory group. Ring enhancement in RCC is reported infrequently, with research suggesting a possible association with inflammation [13, 14, 41, 42]. Chotai et al. [34] reported ring enhancement as an independent predictor of squamous metaplasia. Broad circumferential and thick wall enhancement suggests the potential for abscess development. In our patients, the cystic fluid did not undergo culture testing, precluding the definitive exclusion of infectious complications. Nonetheless, the incidence of pituitary abscesses in RCC cases is notably low [43, 44]. Thick wall enhancement of RCC has been rarely reported in previous studies, but its rarity is considered useful for differentiation from craniopharyngioma [18, 23]. The causative mechanism of thick wall enhancement is unclear; however, it is presumed to be due to inflammation in this study. Mucosal thickening was observed in only one case in our study; therefore, we cannot attribute RCC wall enhancement to paranasal inflammation. In RCC, inflammation occurs spontaneously and often causes ring enhancement.

Basisphenoid enhancement is a recently proposed imaging finding [26]. Originally identified in granulomatous hypophysitis cases, subsequent reports have linked it to ruptured RCC cases [45]. While this enhancement was commonly seen in patients with inflammatory RCC in our study, significant difference was not observed when compared to the non-inflammatory cohort. The prevalence of a post-sellar-type sphenoid sinus could have affected these findings.

Our study has some limitations. First, the epithelial cells of the cyst were not histologically confirmed in some cases in noninflammatory RCC group; thus, it is possible that inflammatory RCC was included in the noninflammatory RCC group. Second, all patients were indicated for surgery; therefore, the prevalence of inflammatory RCC in our cohort differed from that reported in daily practice. Third, the definition of wall enhancement is a shortcoming of this study because thinned normal anterior lobe could be misinterpreted as part of the wall, although differentiation between stretched anterior lobe and enhanced cyst wall is usually easy [9]. Finally, the CT and MRI scanners and protocols were not standardized as they were updated and modified during the nearly 15 years that comprised the study period.

To summarize, inflammatory RCCs often present with central diabetes insipidus preoperatively and tend to have turbid cyst content compared with non-inflammatory RCCs. Furthermore, they exhibited a lower mean ADC value and a wider range of wall enhancement on MRI. Physicians should consider these atypical findings when assessing symptomatic cystic sellar lesions. Further studies are warranted to determine how this information impacts the decision-making of surgeons.
